# Evaluation of differential qPE9-1/DEP1 protein domains in rice grain length and weight variation

**DOI:** 10.1186/s12284-019-0263-4

**Published:** 2019-01-31

**Authors:** Xiangbo Li, Quandan Tao, Jun Miao, Zefeng Yang, Minghong Gu, Guohua Liang, Yong Zhou

**Affiliations:** 1 Jiangsu Key Laboratory of Crop Genetics and Physiology / Key Laboratory of Plant Functional Genomics of the Ministry of Education / Jiangsu Key Laboratory of Crop Genomics and Molecular Breeding, Agricultural College of Yangzhou University, Yangzhou, 225009 China; 2 Jiangsu Co-Innovation Center for Modern Production Technology of Grain Crops, Yangzhou University, Yangzhou, 225009 China

**Keywords:** Rice, Heterotrimeric G protein, Grain size, Yield production

## Abstract

**Background:**

*qPE9–1*/*DEP1*, encoding a G protein γ subunit, has multiple effects on plant architecture, grain size, and yield in rice. The qPE9–1 protein contains an N-terminal G gamma-like (GGL) domain, a putative transmembrane domain, and a C-terminal cysteine-rich domain. However, the roles of each domain remain unclear.

**Results:**

In the present study, we focused on the genetic effects of different domains of qPE9–1 in the regulation of grain length and weight. We generated a series of transgenic plants expressing different truncated qPE9–1 proteins through constitutive expression and clustered regularly interspaced palindromic repeats (CRISPR)/CRISPR-associated protein 9 strategies. Phenotypic analysis indicated that the complete or long-tailed qPE9–1 contributed to the elongation of grains, while the GGL domain alone and short-tailed qPE9–1 led to short grains. The long C-terminus of qPE9–1 including two or three C-terminal von Willebrand factor type C domains effectively repressed the negative effects of the GGL domain on grain length and weight. *qPE9–1*-overexpressing lines in a Wuxianggeng 9 (carrying a *qpe9–1* allele) background showed increased grain yield per plant, but lodging occurred in some years.

**Conclusions:**

Manipulation of the C-terminal length of *qPE9–1* through genetic engineering can be used to generate varieties with various grain lengths and weights according to different requirements in rice breeding. The genetic effects of *qPE9–1*/*qpe9–1* are multidimensional, and breeders should take into account other factors including genetic backgrounds and planting conditions in the use of *qPE9–1*/*qpe9–1*.

**Electronic supplementary material:**

The online version of this article (10.1186/s12284-019-0263-4) contains supplementary material, which is available to authorized users.

## Background

As one of the most important crops worldwide, rice (*Oryza sativa* L.) provides 35%–60% of the world’s dietary calories and is consumed by more than 3 billion people (Fageria, 2007). During the last half-century, global rice production has increased dramatically, primarily because of genetic improvements resulting from the use of semi-dwarfing genes and heterosis to produce hybrid rice. However, an apparent plateau in development has been observed in the last 20 years (Yang and Zhang, 2010).

Grain length not only influences rice grain yield but also affects the physical appearance of the grain and the quality of rice for cooking and eating. Rice grains range from less than 3 mm to more than 11 mm in length (Fitzgerald et al., 2009). The preference for rice varieties with different grain lengths varies among consumer groups (Li et al., 2004). For instance, varieties with long, slender grains are preferred by consumers and cultivators in the USA and most Asian countries. However, short, round grain varieties are popular in Japan, South Korea, and Sri Lanka (Li et al., 2004). Thus, rice grain length has a direct effect on marketability and, hence, commercial success. Grain length also affects 1000-grain weight, one of the yield components of rice, and subsequently determines rice productivity (Tan et al., 2000). To date, more than 60 genes determining rice grain length and weight have been cloned, and several signaling pathways including heterotrimeric G protein, the ubiquitin–proteasome pathway, the mitogen-activated protein kinase signaling pathway, phytohormones and transcriptional regulatory factors, have been investigated (Li et al., 2016b; Xu et al., 2016; Li et al., 2018).

G proteins consisting of G_α_, G_β_, and G_γ_ subunits are involved in a wide range of plant processes including morphological development (Fujisawa et al., 1999; Utsunomiya et al., 2011; Li et al., 2012; Thung et al., 2012; Peng et al., 2018), cell proliferation (Ullah et al., 2001; Chen et al., 2003; Ishida et al., 2014), stomatal control (Wang et al., 2015), abiotic stress (Ma et al., 2015; Yu and Assmann, 2015; Kaur et al., 2018), ion channel regulation (Chakravorty et al., 2011), light perception and protection (Ferrero-Serrano et al., 2018), and responses to phytohormones (Shi et al., 2015; Subramaniam et al., 2016; Zhang et al., 2017). Humans possess 23 G_α_, five G_β_, and 12 G_γ_ subunits (Wettschureck and Offermanns, 2005), while the rice genome has only one G_α_ (*RGA1*), one G_β_ (*RGB1*), and five G_β_ (*RGG1*, *RGG2*, *GS3*, *qPE9–1*, and *GGC2*) genes (Sun et al., 2018). Rice dwarf mutant *d1*, which is defective in *RGA1*, showed defective gibberellin signal transduction and produces small grains (Ashikari et al., 1999; Fujisawa et al., 1999; Ueguchi-Tanaka et al., 2000). *RGB1* knockdown lines had decreased plant heights, and reduced panicle and grain sizes compared with wild-type (Utsunomiya et al., 2011). Thus, *RGA1* and *RGB1* are positive regulators in rice grain size. However, the G_γ_ subunit-encoding genes play diverse roles in grain development.

*GS3* was the first molecularly characterized quantitative trait locus (QTL) for grain size in rice (Fan et al., 2006). Varieties containing a C → A natural mutation in the second exon resulting in loss of the *GS3* allele produce extremely long grains. The GS3 protein is composed of a plant-specific organ size regulation (OSR) domain in the N-terminus, a transmembrane domain, a tumor necrosis factor receptor/nerve growth factor receptor (TNFR/NGFR) family cysteine-rich domain, and a von Willebrand factor type C (VWFC) domain in the C terminus, which function differentially in grain size regulation (Fan et al., 2006; Mao et al., 2010). The OSR domain (also considered a G gamma-like domain, GGL) is both necessary and sufficient to limit grain size, whereas C-terminal TNFR/NGFR and VWFC domains have an inhibitory effect on the OSR function. Deletion of the C-terminal cysteine-rich region leaving most of the OSR domain intact causes plants to produce extremely short grains (Mao et al., 2010).

Previously, we characterized a major rice QTL associated with plant and panicle architecture, *qPE9–1* (Zhou et al., 2009), which is allelic to *DEP1* (Huang et al., 2009). Deletion of part of the C-terminal region at this locus reduced the plant height, and produced small grains and short, erect panicles; the mutation allele, *qpe9–1*, is widely employed in most *japonica* varieties in China. *qPE9–1/DEP1* was also shown to be involved in nitrogen use efficiency in rice (Sun et al., 2014), while we detected a function for *qPE9–1* as a negative regulator in abscisic acid (ABA)-dependent drought-stress responses (Zhang et al., 2015). These data indicate that *qPE9–1*/*DEP1* has multiple effects on rice growth and development, and plays essential roles in yield production.

The qPE9–1 protein contains an N-terminal GGL domain, a putative transmembrane domain, and a C-terminal cysteine-rich domain. To investigate the effects of these domains on grain size regulation, we herein generated a series of transgenic plants expressing different truncated qPE9–1 proteins through constitutive expression and CRISPR/Cas9 (clustered regularly interspaced short palindromic repeats and CRISPR-associated protein 9) strategies.

## Results

### *qPE9–1* acts as a positive regulator of grain size in rice

CRISPR/Cas9 technology has been demonstrated to achieve efficient targeted mutagenesis in transgenic rice (Hu et al., 2016, Ma et al., 2016; Li et al., 2017; Lu et al., 2017), so was used here to specifically disrupt *qPE9–1*. We designed single guide (sg) RNAs targeting the second and fifth exon of *qPE9–1*, and simultaneously transformed them into Nipponbare. More than 20 independent transgenic lines were obtained. Sequencing of PCR-amplified *qPE9–1* genomic DNA from the transgenic plants resulted in the identification of at least 12 independent and effective knockout mutants. Three independent homozygous mutant lines, *qpe9–1#1*, *qpe9–1#2*, and *qpe9–1#3*, were confirmed and grown for agronomic trait examination.

**Fig. 1:**
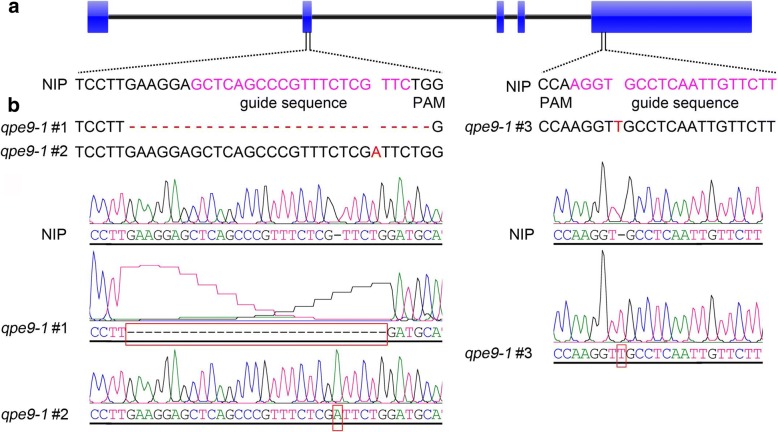
Identification of the three *qpe9–1* mutants generated using CRISPR/Cas9. **a** Two specific target guide RNA sequences located in the second and fifth exons were selected to edit *qPE9–1*. Target guide sequences are marked in pink. **b** The mutation sites of *qPE9–1* in the three mutants, *qpe9–1#1*, *qpe9–1#2*, and *qpe9–1#3*, were confirmed by sequencing. Base variation is shown by the red box

**Fig. 2:**
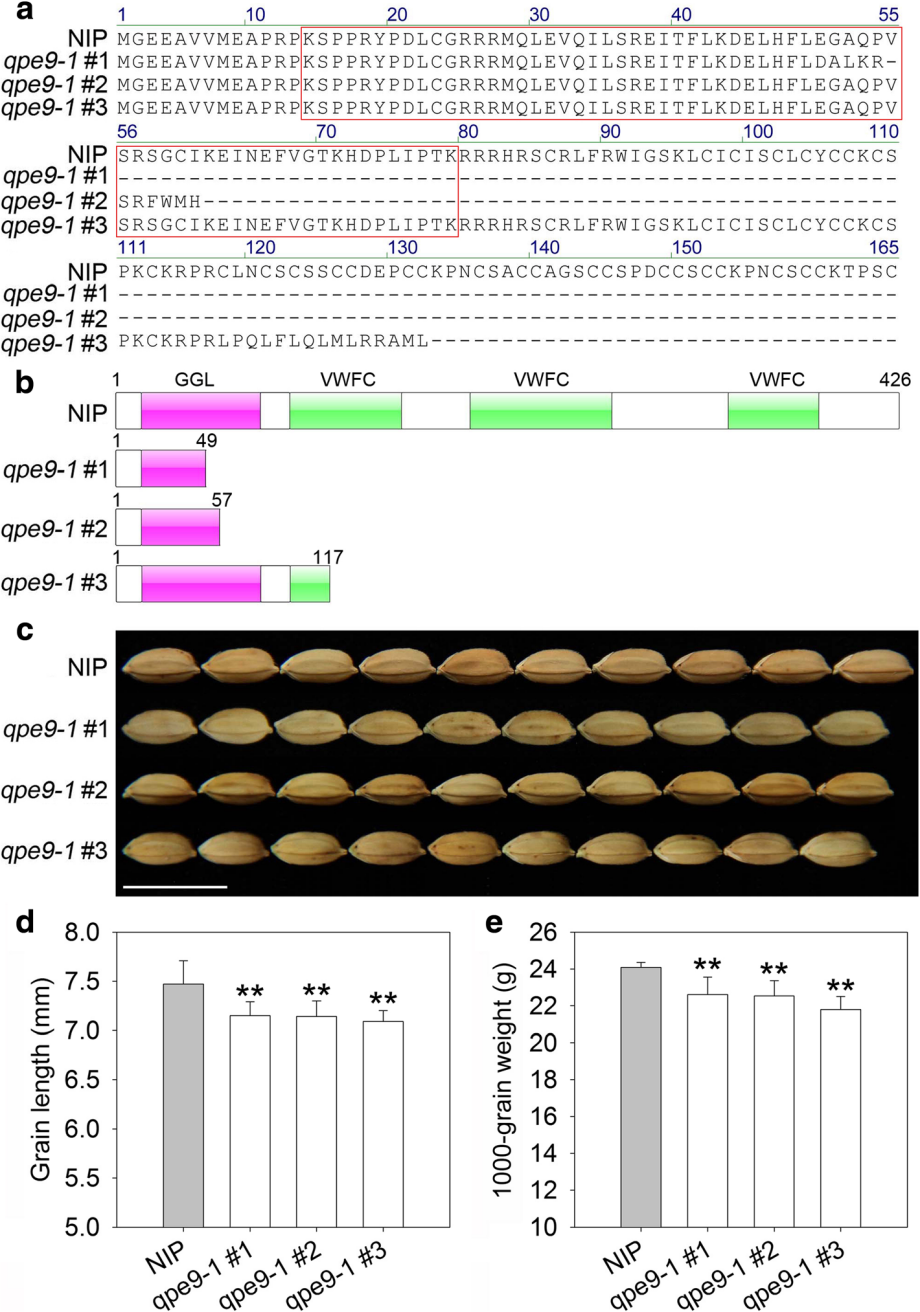
Gene editing of *qPE9–1* led to small grains in the Nipponbare background. **a** Protein sequences of qPE9–1 in the NIP, *qpe9–1#1*, *qpe9–1#2*, and *qpe9–1#3* plants. The GGL domain is shown by red boxes. **b** Structure of the qPE9–1 protein in NIP, *qpe9–1#1*, *qpe9–1#2*, and *qpe9–1#3* plants. The numbers represent the different lengths of the truncated qPE9–1 protein in wild-type plants and the three mutants. **c** Grains of wild-type plants and the three *qpe9–1* mutants. Bar = 1 cm. **d** Comparison of grain length between NIP and the three *qpe9–1* mutants. **e** Comparison of 1000-grain weight between NIP and the three *qpe9–1* mutants. Data are shown as means ± SDs (*n* = 15). Student’s *t*-test: ** *P* < 0.01

The mutation sites of *qpe9–1#1* and *qpe9–1#2* occurred at the second exon, which generated two knockout mutants of *qPE9–1* (Fig. 1). These two mutants lost most of the qPE9–1 protein, which covered part of the GGL domain and all C-terminal VWFC domains (Fig. 2a, b). The average grain lengths of *qpe9–1#1* and *qpe9–1#2* were 7.15 ± 0.14 and 7.14 ± 0.16 mm, respectively, while the wild-type grain length was 7.47 ± 0.24 mm. Thus, *qpe9–1#1* and *qpe9–1#2* plants showed an average 4.37% reduction in grain length (Fig. 2c, d). The 1000-grain weight of *qpe9–1#1* and *qpe9–1#2* plants was significantly decreased by 6.17% and 6.48%, respectively (Fig. 2e). The mutation site in *qpe9–1#3* lines was located in the fifth exon of the target gene, and caused a deletion of the qPE9–1 C-terminal (Fig. 2a, b). The grain length and weight of *qpe9–1#3* were significantly decreased by 5.08% and 9.52%, respectively (Fig. 2d, e). These data show that intact *qPE9–1* acts as a positive regulator of grain length and weight in rice, and that knockout of *qPE9–1* leads to short and small grains.

### Overexpression of different qPE9–1 protein domains generates grains of different length and weight

**Fig. 3:**
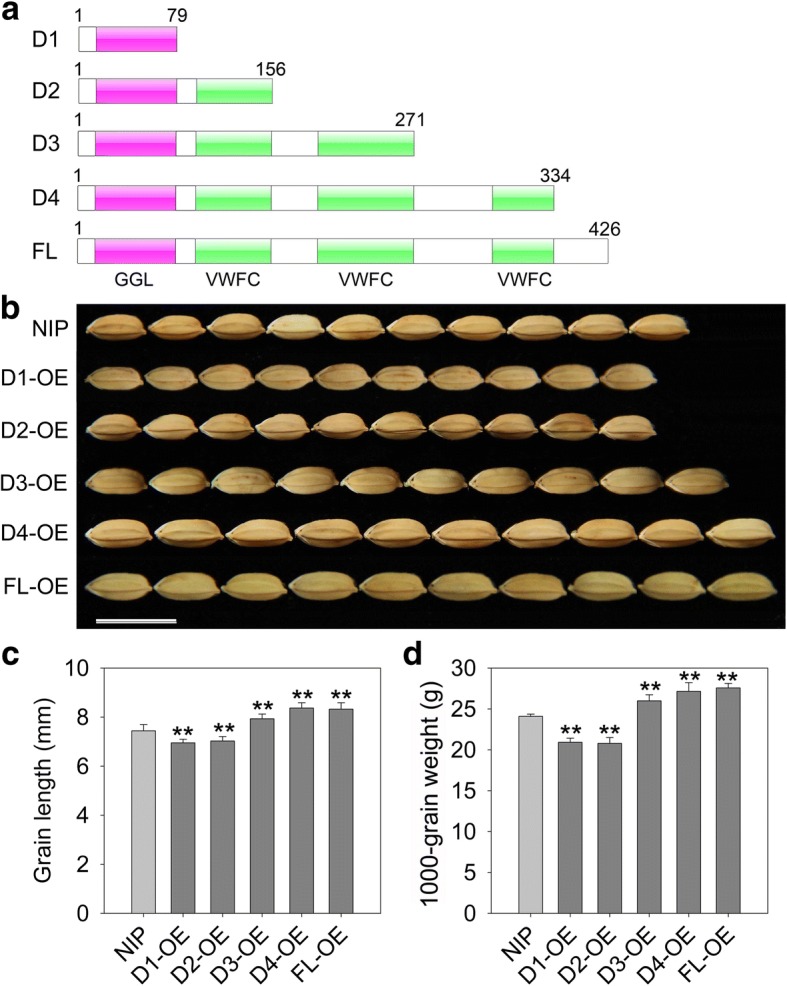
Overexpression of different domains of qPE9–1 generated grains of different lengths and weights. **a** Several constructs covering different domains of qPE9–1. D1 contained the GGL domain and lacked three VWFC domains. D2, D3, and D4 contained the GGL domain and different numbers of VWFC domains. FL, full-length qPE9–1 protein. **b** Comparisons of grains from wild-type and transgenic plants carrying different constructs. Bar = 1 cm. **c** Comparison of grain length between wild-type and overexpressing transgenic plants. **d** Comparison of 1000-grain weight between wild-type and overexpressing transgenic plants. Data are the average of two independent transgenic lines and are shown as means ± SDs (*n* = 30). Student’s *t*-test: ** *P* < 0.01

To further uncover the function of each domain, several constructs covering different domains of qPE9–1 were generated in which the coding sequences were driven by the maize ubiquitin promoter and then delivered to Nipponbare. We developed a set of transgenic plants overexpressing full-length qPE9–1 (FL), truncated qPE9–1 without all three VWFC domains (D1), truncated qPE9–1 without the second and third VWFC domains (D2), truncated qPE9–1 without the third VWFC domain (D3), and truncated qPE9–1 without part of the C-terminus (D4) (Fig. 3a). At least eight independent transgenic individuals for each construct were obtained. All T_1_ families were grown and screened with single-copy transferred DNA insertion using hygromycin resistance as the marker. Seeds from a single T_1_ plant were harvested, then transgenic lines of the T_2_ generation were grown, and homozygous lines (namely, D1-OE, D2-OE, D3-OE, D4-OE, and FL-OE) for each construct were selected for phenotypic observation.

As shown in Fig. 3b, the transgenic plants overexpressing the full-length cDNA sequence of *qPE9–1* produced larger and heavier grains than wild-type. Compared with Nipponbare, the grain length and weight of FL-OE transgenic plants increased by 11.36% and 14.44%, respectively (Fig. 3c, d). These results further confirm that *qPE9–1* is a positive regulator of grain length and weight in rice. D4-OE plants also produced enlarged grains (+ 11.96% in grain length and + 12.71% in 1000-grain weight) which were similar to those of FL-OE transgenic plants. However, overexpression of the D3 construct had only a mild effect on grain size increase (6.13% in grain length and 7.88% in 1000-grain weight) (Fig. 3b).

The transformants overexpressing the truncated qPE9–1 protein lacking the second and third VWFC domains produced smaller grains (Fig. 3b). We observed a substantial decrease in grain length (− 5.99%) and 1000-grain weight (− 13.70%) in D2-OE transgenic plants compared with those of Nipponbare (Fig. 3c, d). Moreover, transgenic plants carrying the D1 construct overexpressing the GGL domain also exhibited reduced grain length (− 6.97%) and weight (− 13.17%) (Fig. 3). This suggested that the N-terminal GGL domain of qPE9–1 negatively regulates grain length and weight, and that C-terminal VWFC domains may inhibit the effects of the N-terminal to promote grain development.

### Constitutive expression of *qPE9–1* results in higher grain yield per plant in an existing high-yield variety

1000-grain weight is one of the determinants of grain yield in rice. In view of the large effects of *qPE9–1* on grain length and weight, we wondered whether *qPE9–1* could be used to further increase grain yield in an existing high-yield variety. A construct containing full-length *qPE9–1* cDNA driven by the maize ubiquitin promoter was transformed into Wuxianggeng 9 (WXG9, carrying a *qpe9–1* allele), a high-yield fragrant variety of rice, which was widely cultivated in the south of Jiangsu Province, China. More than 20 independent transgenic lines were obtained, and three T_8_ generation homozygous lines (WXG9-OE1, WXG9-OE2, and WXG9-OE3,) were grown for a detailed phenotypic examination.

**Fig. 4:**
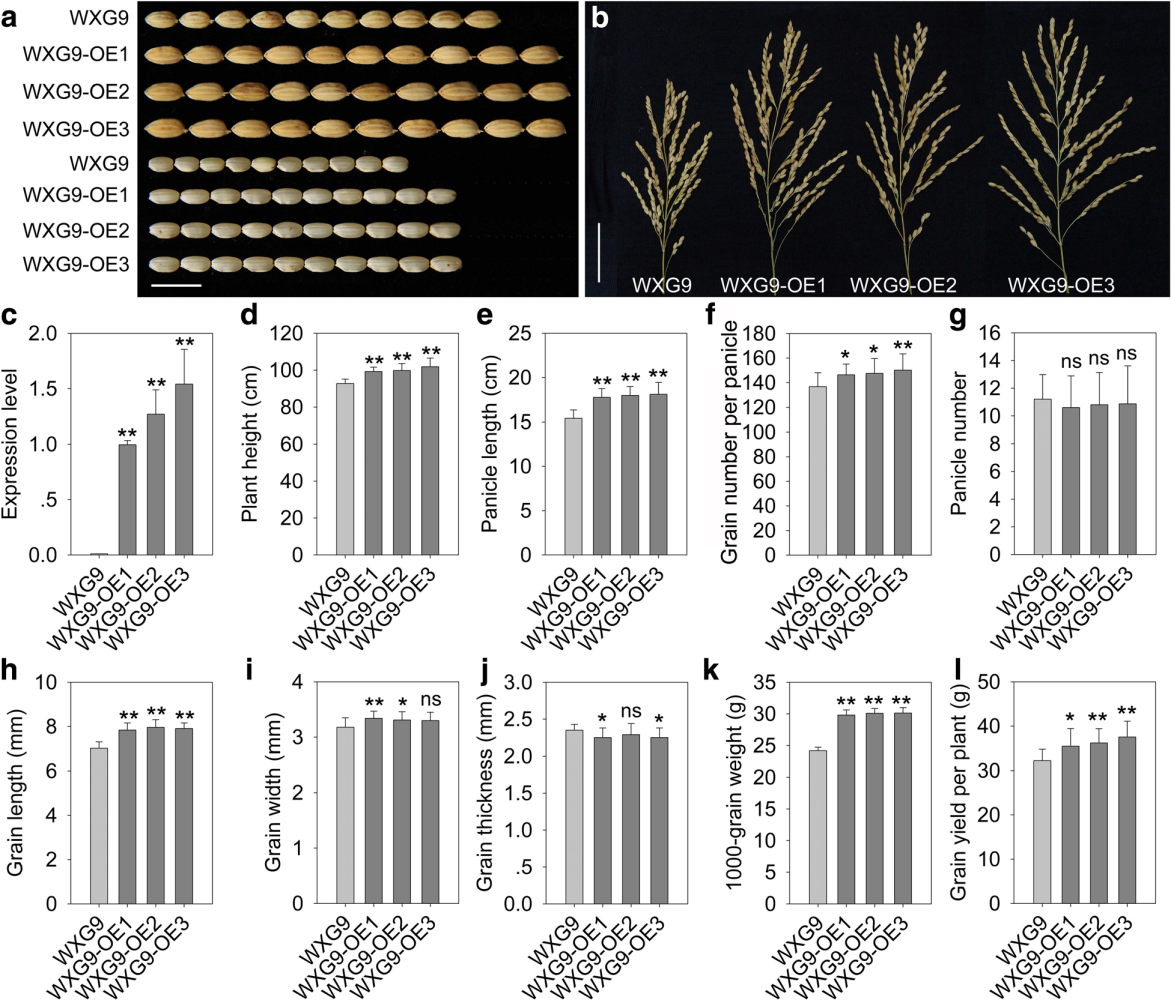
Overexpression of *qPE9–1* in high-yield rice Wuxianggeng 9 affected multiple agronomic traits. **a** Grains and brown rice of WXG9 and three overexpressing lines, WXG9-OE1, WXG9-OE2, and WXG9-OE3. Bar = 1 cm. **b** Panicles of WXG9 and three overexpressing transgenic lines. Bar = 5 cm. **c-l** Comparisons of *qPE9–1* expression level (c), plant height (d), panicle length (e), grain number per panicle (f), panicle number per plant (g), grain length (h), grain width (i), grain thickness (j), 1000-grain weight (k), and grain yield per plant (l), between WXG9 and three overexpressing lines. Data are shown as means ± SDs (n = 15). Student’s *t*-test: *P < 0.05; **P < 0.01; ns, not significant

qPCR indicated that *qPE9–1* expression levels were significantly elevated in overexpressing lines compared with wild-type (Fig. 4c). At maturity, the three *qPE9–1*-overexpressing lines were significantly taller than wild-type (Fig. 4d). WXG9-OE1, WXG9-OE2, and WXG9-OE3 plants also had elongated panicle lengths and increased panicle sizes (Fig. 4b, e, and f). The panicle number per plant of overexpression lines was slightly decreased, but the difference was not significant (Fig. 4g). Additionally, the transgenic lines exhibited an obviously increased grain size (Fig. 4a, h, i, and j), and the 1000-grain weight of the three lines was increased by 23.27%, 24.46%, and 24.57%, respectively (Fig. 4k). Thus, the grain yield per plant increased by 9.90%, 12.19%, and 16.35%, respectively, compared with wild-type (Fig. 4l). These data suggest that *qPE9–1* promotes plant growth and improves grain yield per plant in an existing high-yield variety background.

## Discussion

Heterotrimeric G proteins are universal signaling components in eukaryotes, and their regulatory properties have been thoroughly investigated in animals (Urano et al., 2012; Urano et al., 2013; Bender and Zipfel, 2018). The classic heterotrimers consist of three different subunits, designated G_α_, G_β,_ and G_γ_. In mammalian cells, activation of G-protein-coupled receptors (GPCRs) upon ligand binding leads to GDP/GTP exchange and the activation of G_α_, which causes its dissociation from the G_βγ_ dimer. Disassociated G_α_ and G_βγ_ then activate their own downstream signaling elements to regulate various biological processes. The situation differs in plants, where the canonical G_α_, G_β,_ and G_γ_ are encoded by a small number of genes (Bender and Zipfel, 2018). Because of the spontaneous guanine nucleotide exchange activity of plant G_α_ subunits and the absence of plant GPCRs, the molecular mechanism of heterotrimeric G proteins for plant growth and development departs from the accepted animal paradigm (Trusov and Botella, 2016).

The function of G_γ_ was initially thought to be restricted to anchoring G_βγ_ dimers to the membrane. In plants, G_γ_ subunits can be divided into three clades, type A, type B, and type C, which exhibit extraordinary structural diversities and differences compared with their mammalian counterparts (Xu et al., 2016). Type A G_γ_ subunits are very small (fewer than 100 amino acids), but contain all the conserved features of mammalian G_γ_ proteins. Type B G_γ_ subunits lack the CaaX motif for isoprenylation (Trusov. et al., 2012; Urano et al., 2012). Type C G_γ_ subunits belong to a novel class that is widespread throughout the plant kingdom but is nonexistent in animals (Sun et al., 2014). They have an N-terminal GGL domain, a weakly predicted transmembrane helix in the central region, and a long C-terminal cysteine-rich region that consists of VWFC and TNFR domains. In rice, the three homologs, GS3, qPE9–1/DEP1, and GGC2, belong to type C. Mao et al. (2010) showed that the OSR (GGL) domain of GS3 is both necessary and sufficient to function as a negative regulator. Loss-of-function of OSR results in long grains, while C-terminal TNFR/NGFR and VWFC domains have an inhibitory effect on the OSR function. However, the roles of each domain of qPE9–1/DEP1 remained unclear.

In this study, we generated several homozygous knockout mutants of *qPE9–1* in the Nipponbare background using CRISPR/Cas9. All three independent mutants, *qpe9–1#1*, *qpe9–1#2*, and *qpe9–1#3*, lost most of the qPE9–1 protein and showed a decreased grain length and weight (Fig. 2). We also obtained *qPE9–1*-overexpressing lines in Nipponbare and Wuxianggeng 9 backgrounds, which all produced larger and heavy grains. These results confirmed that *qPE9–1* is functional and plays a positive role in rice grain length and weight. Recently, Sun et al. (2018) reported that DEP1 and GGC2, either individually or in combination, increase grain length when complexed with RGB1, which is consistent with our results. We further generated a series of transgenic lines overexpressing truncated *qPE9–1* segments covering various protein domains. As shown in Fig. 3, the transformants overexpressing D1 and D2 constructs produced shorter and lighter grains, while those overexpressing D3 and D4 constructs had longer and heavier grains. The grain length of D4-OE plants was similar to that in transformants overexpressing full-length *qPE9–1*. This indicated that the N-terminal GGL of qPE9–1 negatively regulates rice grain length and weight, and that its inhibitory effect is suppressed by the C-terminal VWFC domains. Elevated levels of truncated qPE9–1 protein containing two or three VWFC domains were shown to increase grain length and weight, while only one VWFC domain was insufficient to modify the inhibition of the GGL domain on grain size. This suggested that the cysteine-rich C-terminal tail of qPE9–1 is critical in the regulation of grain length and weight.

The N-terminal GGL and C-terminal VWFC domains of qPE9–1 exert antagonistic effects in grain length and weight regulation, which is similar to the head-to-tail model obtained from GS3 (Mao et al., 2010). Sun et al. (2018) further proposed a genetic model depicting the pathway of the G proteins in grain size regulation. In this model, qPE9–1/DEP1 and GGC2 positively regulate grain size, while GS3 alone has no effect. However, the competitive interaction of GS3 with RGB1 disrupts RGB1–qPE9–1/DEP1 and RGB1–GGC2 dimers, resulting in short grains. This model also provides a possible explanation for the fact that the overexpressed GGL domain of qPE9–1 decreased the grain length and weight in this study. However, it was also demonstrated that both GS3 and qPE9–1/DEP1 play negative roles in the regulation of grain size by promoting activity of the OsMADS1 transcription factor (Liu et al., 2018). Previously, Li et al. (2016a) assessed the genetic effects of *qPE9–1/DEP1* using CRISPR/Cas9, and found that the grains of two knockout mutants were significantly lighter than those of wild-type plants. Taken together, we believe that *qPE9–1/DEP1* is a positive regulator of rice grain length and weight.

Suppression of *RGB1* expression causes dwarfism and small grains (Utsunomiya et al., 2011), so *RGB1* is also considered a positive regulator of rice grain size. RGB1 forms a dimer with qPE9–1/DEP1 and GGC2 to promote grain growth (Sun et al., 2018). However, *RGB1* mutants have not been identified in rice, and it is generally agreed that *RGB1* knockout is lethal. We obtained several transgenic lines of *RGB1* in Nipponbare (carrying the *qPE9–1/DEP1* allele) and Wuyungeng 8 (carrying the *qpe9–1/dep1* allele) backgrounds, using RNA interference-mediated gene silencing, which resulted in decreased plant height and reduced grain size (data not shown). It was recently reported that *RGB1* overexpression led to small grains (Liu et al., 2018). However, we observed no obvious phenotypic variation in grain size in Nipponbare plants overexpressing *RGB1* (data not shown). The actual function of *RGB1* in rice grain size regulation is therefore unclear, although it is possible that RGB1 protein in wild-type is sufficient to form a dimer with G_βγ_ to regulate plant and grain growth.

In the present study, we also overexpressed *qPE9–1* in a high-yield rice variety, WXG9, which carries *qpe9–1* and produces short and erect panicles. As expected, homozygous and stable transgenic lines exhibited increased plant height and panicle length, as well as enlarged grain size (Fig. 4). The grain yield per plant of the overexpression lines was significantly increased compared with WXG9, although the panicle number per plant was slightly decreased. However, during 2009–2016, we observed frequent lodging in *qPE9–1*-overexpressing lines in WXG9, especially in conditions of high nitrogen input and high plant density (data not shown). The introgression of *qPE9–1* is known to significantly increase plant height (Zhou et al., 2009; Yi et al., 2011). Because plant height is negatively associated with the rice anti-lodging trait, the loss-of-function *qpe9–1* allele appears to enhance the rice anti-lodging capacity by reducing plant height.

The genetic effects of *qPE9–1*/*DEP1* on grain yield are controversial. Huang et al. (2009) indicated that the mutation allele *qpe9–1*/*dep1* increased grain yield per plant. However, several studies reported that *qpe9–1* has negative or background-dependent roles on grain yield per plant (Chen et al., 2006; Zhou et al., 2009; Fumio et al., 2011; Yi et al., 2011; Lu et al., 2013). In fact, the *qpe9–1*/*dep1* allele is only used in *japonica* varieties, while the *qPE9–1*/*DEP1* allele is widely employed in *indica* varieties. It remains unclear why a gene that decreases grain yield per plant is widely used in *japonica* rice production. Previously, we proposed that *qpe9–1* is a double-edged sword allele in rice breeding, in that it shapes an ideal plant architecture but has negative effects on the individual yield of the plant (Zhou et al., 2009). Rice varieties carrying *qpe9–1* usually produce short, erect panicles and leaves. This compact plant architecture is not only beneficial in improving ventilation and light penetration, but is also suitable for close planting. In the present study, WXG9 overexpression lines produced larger panicle sizes compared with wild-type (Fig. 4b, f). Previously, we reported that *qPE9–1* had no effect on the grain number per panicle (Zhou et al., 2009), although Huang et al. (2009) indicated that the *qpe9–1*/*dep1* allele significantly increased grain number per panicle. Moreover, a loss-of-function mutation of *DENSE PANICLE 1*, which is allelic to *qPE9–1/DEP1*, caused semi-dwarfness and a slightly increased number of spikelets (Fumio et al., 2011), while the deletion of *qPE9–1/DEP1* in *indica* rice via CRISPR/Cas9 resulted in small grains and reduced panicle sizes (Wang et al., 2017). Taken together, the genetic effects of *qPE9–1*/*qpe9–1* appear to be complicated, and may change according to different genetic backgrounds and field conditions.

## Conclusions

In the present study, we generated a series of transgenic plants expressing different truncated qPE9–1 protein constructs through constitutive expression and CRISPR/Cas9 strategies. Phenotypic analysis indicated that full-length or long-tailed qPE9–1 produced long and heavy grains, while the GGL domain alone and short-tailed qPE9–1 produced short and light grains. Moreover, the long C-terminus of qPE9–1 including two or three C-terminal VWFC domains effectively repressed the negative effects of the GGL domain on grain length and weight. Manipulation of the C-terminal length of qPE9–1 through genetic engineering can therefore be used to generate varieties with various grain lengths and weights according to different requirements in rice breeding. Overexpression of *qPE9–1* increased grain size and yield in the Wuxianggeng 9 background, but led to lodging in some years. Breeders should take into account other factors including genetic backgrounds and planting conditions in the use of *qPE9–1*/*qpe9–1.*

## Methods

### Vector construction and transformation

Two specific target guide RNA sequences located in the second and fifth exons were selected to generate *qPE9–1* mutants. The final fragment was inserted into a CRISPR/Cas9 system (Baige Biotech, Hangzhou City, China) in which the Cas9 destination vector was driven by the maize ubiquitin promoter for expression in rice, and sgRNA expression was driven by the U6 promoter. The CRISPR/Cas9 construct was transformed into Nipponbare via *Agrobacterium tumefaciens*-mediated transformation. The different variations of *qPE9–1* obtained from CRISPR/Cas9 editing were sequenced with specific primers (Table S1). The homozygous transgenic lines of mutants were used for functional analysis.

The full and partial coding regions of *qPE9–1* were amplified from Nipponbare cDNA, and inserted into the p1301UbiNOS vector to generate several overexpression constructs which were driven by a constitutively expressed maize ubiquitin promoter (Zhou et al., 2009). All constructs were transferred into Nipponbare and Wuxianggeng 9 by *A. tumefaciens*-mediated transformation.

### Plant growth conditions

Forty plants of each line were grown in the experimental field of Yangzhou University (E119°25′/N32°23′) in the summer of 2017 for molecular and phenotypic evaluation. Ten seedlings (approximately 4-weeks-old) per row were transplanted with a distance of 17.0 cm between plants and 23.3 cm between rows. After transplanting, they were fertilized with nitrogen (225 kg ha^− 1^ as urea), phosphorus (50 kg ha^− 1^ as single superphosphate), and potassium (60 kg ha^− 1^ as KCl). Field management and disease and pest control followed standard procedures to prevent yield loss during the growth period.

### Phenotypic evaluation

At maturity, several traits of wild-type and transgenic lines were measured to investigate the roles of each domain of qPE9–1. Plant height was measured from the ground surface to the tallest panicle. Panicles of the main stem were selected for measuring panicle length and counting the grain number per panicle. Panicle number per plant was the number of effective panicles with 10 or more grains. The length, width, and thickness of grains were measured using vernier calipers after harvesting and storage at 37 °C for at least 2 weeks. The weight of 100 plump grains was obtained and then converted to the 1000-grain weight. All grains of one plant were weighed to measure the grain yield per plant.

### RNA preparation and reverse transcription quantitative PCR (qPCR)

Total RNA was extracted with an RNA Prep Pure Kit (Tiangen Biotech, Beijing City, China) in accordance with the manufacturer’s instructions and then treated with DNase to digest any genomic DNA. cDNA was synthesized from 1 μg of total RNA using a reverse transcription kit (Tiangen Biotech).

qPCR was performed in a total volume of 25 μl, which consisted of 2 μl of cDNA, 0.2 mM of each primer, and 12.5 μl of 2× SYBR Green PCR Master Mix (Takara Bio, Shiga, Japan). The qPCR assay was conducted using a qPCR system (ViiA7, Applied Biosystems, Foster City, USA) using the following program: 95 °C for 3 min followed by 40 cycles of 94 °C for 30 s, 55 °C for 30 s, and 72 °C for 40 s. qPCR analysis was carried out using the rice *actin* gene (LOC_Os03g50885) as an internal control. Data are presented as the mean values of three replicates. Relative gene expression was calculated using the 2^−ΔΔCT^ method. qPCR primers are listed in Additional file 1: Table S1.

### Statistical analysis

All numerical data are presented as the means ± SDs (error bars indicate the standard deviations of the means). Statistical analyses were carried out using Excel (Microsoft, USA) and SigmaPlot software (Systat, USA). The differences between transgenic and wild-type plants were determined using the Student’s *t*-test (*, *P* < 0.05; **, *P* < 0.01; ns, not significant).

## Additional file

Additional file 1: **Table S1.** Primers used in this study. (DOC 32 kb)
