# Supplementary material for: Evaluation of differential qPE9-1/DEP1 protein domains in rice grain length and weight variation
Source: Rice (N Y). 2019 Jan 31;12:5. doi: 10.1186/s12284-019-0263-4 (PMC6357212; doi:10.1186/s12284-019-0263-4)
Supplement: Supplementary file 1 — Table S1. Primers used in this study. (DOC 32 kb) [file 12284_2019_263_MOESM1_ESM.doc]

Table S1. Primers used in this study.

| Primer name | Sequence (5'-3') |
| --- | --- |
| FL-OE F | AAAAGGATCCGGGGTGGTTCTGAGTTGG |
| FL-OE R | AAAAACTAGTCGGTTCAACCTCGTCTCATA |
| D1-OE F | AAAGGATCCATGGGGGAGGAGGCGGTGGTG |
| D1-OE R | AAAACTAGTTCACTTTGTTGGTATTAGTGG |
| D2-OE R | AAAACTAGTTCAGTTAGGTTTACAGCATGA |
| D3-OE R | AAAACTAGTTCAGCAGCTTGGAAGGCCACAG |
| D4-OE R | AAAACTAGTTCAGGAGCATTTGAAGATG |
| qPE9-1 cas9-1 F | ggcaGCTCAGCCCGTTTCTCGTTC |
| qPE9-1 cas9-1 R | aaacGAACGAGAAACGGGCTGAGC |
| Cas9 seq-1F | TCTAGCTCAAGGAGGAAAT |
| Cas9 seq-1R | CTGAGGCAGAGTTATAGGC |
| qPE9-1 cas9-2 F | ggcaAAGAACAATTGAGGCACCT |
| qPE9-1 cas9-2 R | aaacAGGTGCCTCAATTGTTCTT |
| Cas9 seq-2F | GTCTGAAAGTGCCTCTAGTCCC |
| Cas9 seq-2R | GTTTGCAGCAAGAAGGGGTC |
| qPE9-1-qPCR-F | GGAGGAGGCGGTGGTGAT |
| qPE9-1-qPCR-R | CACCGAAAAAGACGGCAAG |
